# Role of Diet and Nutritional Supplements in Parkinson's Disease Progression

**DOI:** 10.1155/2017/6405278

**Published:** 2017-09-10

**Authors:** Laurie K. Mischley, Richard C. Lau, Rachel D. Bennett

**Affiliations:** ^1^Bastyr University Research Institute, 14500 Juanita Dr. NE, Kenmore, WA 98028, USA; ^2^Oregon State University, 101 Milam Hall, Corvallis, OR 97331, USA

## Abstract

**Objectives:**

The goal of this study is to describe modifiable lifestyle variables associated with reduced rate of Parkinson's disease (PD) progression.

**Methods:**

The patient-reported outcomes in PD (PRO-PD) were used as the primary outcome measure, and a food frequency questionnaire (FFQ) was used to assess dietary intake. In this cross-sectional analysis, regression analysis was performed on baseline data to identify the nutritional and pharmacological interventions associated with the rate of PD progression. All analyses were adjusted for age, gender, and years since diagnosis.

**Results:**

1053 individuals with self-reported idiopathic PD were available for analysis. Foods associated with the reduced rate of PD progression included fresh vegetables, fresh fruit, nuts and seeds, nonfried fish, olive oil, wine, coconut oil, fresh herbs, and spices (*P* < 0.05). Foods associated with more rapid PD progression include canned fruits and vegetables, diet and nondiet soda, fried foods, beef, ice cream, yogurt, and cheese (*P* < 0.05). Nutritional supplements coenzyme Q10 and fish oil were associated with reduced PD progression (*P* = 0.026 and *P* = 0.019, resp.), and iron supplementation was associated with faster progression (*P* = 0.022).

**Discussion:**

These are the first data to provide evidence that targeted nutrition is associated with the rate of PD progression.

## 1. Introduction

Epidemiological studies have shown that consumption of green tea, coffee, and blueberries and dairy avoidance are associated with reduced likelihood of being diagnosed with Parkinson's disease (PD) [1–4]. Food which protects does not necessarily treat and patients already diagnosed want to know “Does my diet and/or lifestyle affect the course of my disease?”

PD is a slowly progressing disease, so disease-modification trials require long follow-up periods. The heterogeneity of the disease requires enrollment of large populations, both of which increase the expense of clinical trials. Motor symptoms are now known to occur late in the disease and thus may not be an ideal outcome measure for protection, although biomarkers of early disease are lacking. Further complicating matters, results of efficacy (or lack thereof) seen in the controlled, ideal environment of a randomized controlled trial, may or may not translate to effectiveness in a real-life setting. To circumvent these issues, a study was designed to ask patients directly about their food choices and use of supplements. The positive deviance approach uses disease heterogeneity to our advantage, permitting the identification of individuals progressing at a substantially slower or faster rate of PD progression [5]. The goal of this study was to describe whether modifiable aspects of lifestyle are associated with PD symptom severity and progression.

## 2. Materials and Methods

“Complementary and alternative medicine in PD (CAM Care in PD)” is a pragmatic, prospective observational study that was designed to accomplish the task. The questionnaire was designed by the PI (LKM) based on her experience as a nutritional neuroepidemiologist, clinical trialist, and physician specializing in PD. A study webpage, hosted by Bastyr University, provided automated access to participation. In addition to recruitment via social media, IRB-approved recruitment cards were distributed at PD support groups and to colleagues at neurology conferences.

**Figure 1:**
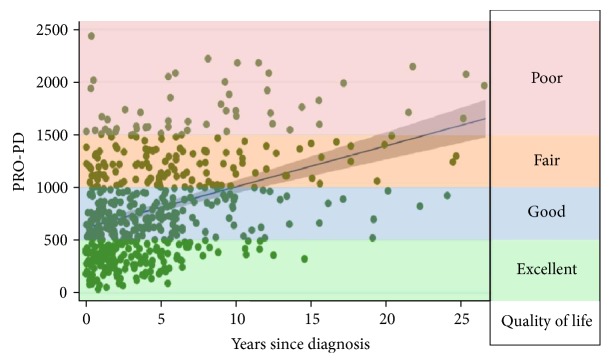
The PRO-PD score is the sum of 33 motor, mood, and other nonmotor symptoms common in PD. Higher scores represent either more symptoms or greater symptom severity of a few symptoms. Lower PRO-PD scores correlate with better social, emotional, and physical quality of life [7].

The primary outcome measure was an assessment tool, patient-reported outcomes in PD (PRO-PD), designed to assess PD severity. The PRO-PD consists of 33 common PD symptoms, and the participant is asked to move the tab on a slider bar according to symptom severity. The left side of the bar always represents optimal health (lack of symptom), and the right side of the bar always represents maximum symptom severity. The PRO-PD score is the cumulative score of each of the symptoms, each of which is assigned a value 0–100, resulting in a continuous outcome measure that increases over time. In a cross-sectional analysis, PRO-PD scores correlated with disease duration (*r* = 0.388, *P* < 0.000), total UPDRS (*r* = 0.446, *P* = 0.008), patient-assessed Hoehn and Yahr (HY) (*r* = 0.636, *P* < 0.000), PDQ-39 (*r* = 0.763, *P* < 0.000), PROMIS Global quality of life question (*r* = −0.744, *P* < 0.000) (Figure 1), and the Timed Up and Go (TUG) (*r* = 0.457, *P* < 0.006). The PRO-PD nonmotor subscore correlated with Nonmotor Symptom Scores (NMSS) (*r* = 0.911, *P* < 0.000) [6].

Individuals with all forms of parkinsonism were invited to participate in the CAM Care in PD study. While the CAM Care in PD study is designed to be longitudinal, this initial analysis is cross-sectional, as more time is needed before there will be adequate sample size for a robust longitudinal analysis. In the meantime, a cross-sectional study adjusted for years since diagnosis can still offer insights into the association between PD severity and diet and lifestyle factors. For this cross-sectional analysis, only baseline data from individuals with a self-reported diagnosis of idiopathic PD were used. PD severity is defined by the cumulative PRO-PD score, and PD progression is defined by the PRO-PD score adjusted for years since diagnosis.

A food frequency questionnaire (FFQ) was developed to quantify dietary intake. The FFQ used in this study was created to be pragmatic and meet the needs of this study by drawing on limitations and successes of other nutritional intake questionnaires. Participants were asked to estimate their intake of foods, on average, over the prior six months. For all dietary variables, participants were given 10 options for rating consumption frequency, ranging from “never” to “5-6 times daily.” Other variables chosen were based on incidence data as well as on biological and clinical relevance. Participants were directed to “Please mark the box if you have taken any of the following consistently over the past 6 months” as well as to identify lifestyle choices that they had been engaged in consistently for the last 6 months. All supplement and behavior variables were recorded as binary variables as people either reported using specific supplements/lifestyles or did not.

Multiple linear and logistic regression models were used to examine the association between diet, lifestyle factors, and PD severity, with PRO-PD scores used as the outcome variable. Food frequency questionnaire data is ordinal; due to the relatively large number (10) of consumption frequencies that were offered as options, it was analyzed as a continuous variable. All models controlled for factors known to heavily influence PD severity including age, years since diagnosis, and gender. Additionally, a second model was created that also incorporated income, which may be associated with lifestyle and access factors that may affect PD severity. All statistical work was done using Stata Version 11 (College Station, TX) with alpha set to 0.05. No adjustments were made for multiple comparisons to avoid increasing the risk of type II errors, and the failure to detect an association that is present was a priority for this observational study [8].

## 3. Results and Discussion

Of the 1307 participants with parkinsonism, 1053 were identified as having a diagnosis of idiopathic PD and were thus available for analysis. The average age of participants was 63 years, with an average 5.2 years since diagnosis. Accordingly, the majority of the study participants were in HY stages 1–3 (93.5%). While gender and income were evenly distributed across the study population, there was very little ethnic diversity (Table 1).

### 3.1. Dietary Variables

Using the food frequency questionnaire, the results of this analysis suggest that a plant- and fish-based diet is associated with the lowest PD severity score (Table 2). Fresh vegetables, fresh fruit, nuts and seeds, fish, olive oil, wine, coconut oil, fresh herbs, and the use of spices were all associated with statistically significant lower rates of disease progression.

These foods largely comprise the Mediterranean diet, which has been associated with reduced PD incidence and later age of diagnosis [9]. Likewise, there is evidence that this diet decreases risk and progression of Alzheimer's disease, a related neurodegenerative disorder [10–12]. Only fresh herbs lost statistical significance after adjusting for income, suggesting that fresh herbs may be a luxury item afforded to those in the higher-income brackets.

Ice cream, cheese, and yogurt intakes were associated with higher rates of PD progression (Table 2). Dairy has been repeatedly associated with PD incidence [13–16], and this is the first study to demonstrate an association between dairy consumption and an increased rate of PD progression. In this cohort, neither milk nor butter are associated with PD progression. Previous studies suggested the association was strongest for milk, ice cream, and cream [16], which was replicated with the Greek study [17]; the latter study suggesting yogurt was not associated with increased risk of PD incidence. These data contradict the findings on yogurt from the Greek study but do support the associations with cream. Self-reported dietary intake is notoriously difficult to assess [18], and it may be that the study participants are underestimating milk and butter consumption. For example, it is difficult to estimate how much milk and butter contribute to foods like mashed potatoes and baked goods.

There may be several mechanisms responsible to explain the association between PD progression and dairy consumption:

1. Dairy intake lowers uric acid [19]. Uric acid quenches peroxynitrite in the CNS, and low uric acid levels are associated with greater PD incidence and faster PD progression [20].
2. Diary consumption is associated with insulin resistance [21]. There is a growing body of evidence that PD and other neurodegenerative diseases are a form of “type III diabetes” [22].
3. Lactose intolerance, occurring when the enzyme, lactase, that digests the milk sugar decreases with age, is especially common in individuals of African, Asian, Hispanic, and Native American decent [23]. Consuming dairy in the absence of sufficient lactase may contribute to intestinal inflammation and intestinal permeability.
4. Presence of a neurotoxic component or contaminant, for example, pesticides, may be present in dairy [23].
5. Introduction of bovine microbiota, facilitating seeding of methanogenic organisms, leads to the development of methane-dominant small intestinal bacterial overgrowth (SIBO) and other forms of abnormal intestinal flora [24–26].

Consumption of canned fruits and vegetables was a strong predictor of PD progression. Initially thought to be associated with socioeconomic status, the association remained after adjusting for income. Bisphenol A (BPA) is used extensively worldwide in the inner coating of food cans, and there is evidence that BPA contaminates foods stored in the cans. BPA is a well-established endocrine conductor associated with obesity, and more recent evidence suggests that it is an energy balance disruptor [27]. Additionally, aluminum is an established neurotoxicant, and the aluminum content of the cans may be contributing to the association [28].

The association with fried foods may be related to lipid peroxidation resulting from the well-established increase in reactive oxygen species (ROS) observed in PD. Lipid peroxidation results in the production of aldehydes, such as acrolein, that bind covalently with thiol groups of proteins, leading to protein aggregation and dysfunction [29]. In the PD substantia nigra, acrolein accumulates in dopaminergic neurons, modifies alpha-synuclein, and inhibits proteasome activity [30]. Accordingly, in recent years, there has been a call to develop new PD treatments that target lipid peroxidation [31].

Soda, specifically diet soda, was also associated with a faster rate of PD progression. Soda is a sugar-sweetened beverage associated with additional caloric intake and obesity [32], which was also associated with PD progression in this study (Table 3). As was discussed in the section on dairy, there is growing evidence that PD might be considered type 3 diabetes. The association with PD progression was higher for diet soda than regular soda. Following consumption, aspartame is metabolized to phenylalanine, aspartic acid, and methanol. The increase in phenylalanine and aspartic acid interferes with the transport of serotonin and dopamine to the brain, increases neuronal hyperexcitability, and leads to degeneration in astrocytes and neurons [33, 34].

The association between beef and PD progression is congruent with traditional epidemiologic research demonstrating an association between beef consumption and PD incidence [2]. Beef and pork, the most frequently consumed mammals in the Western diet, have several things in common, including a high-fat content and slow intestinal transit time. That intake of pork which was not statistically significantly associated with worse prognosis suggests that future research should be directed toward variables unique to beef, such as the higher iron content. Recent research suggests that alpha-synuclein in the enteric nervous system is associated with immune cell activation [35]; as both milk and meat from bovine sources have been linked to incidence and progression of PD, cross-reactivity between common antigens in the enteric nervous system should also be considered [36]. It is well known that dietary protein competes with levodopa for intestinal absorption and it is possible the high protein content of beef and dairy may not be affecting the disease, but making the medication less effective.

### 3.2. Nutritional Supplements

Of all the nutritional supplements studied, only coenzyme Q10 and fish oil were associated with statistically significant reduced rates of PD progression (Table 3). The association between coenzyme Q10 and PD progression disappeared after adjusting for income, which was not unexpected given the high cost of the supplement and lack of third-party reimbursement. In a clinical case series, patients with PD had significantly greater odds of deficiency in coenzyme Q10 status compared to controls (OR: 4.7–5.4; 95% CI: 1.5–17.7; *P* = 0.003–0.009). The proportion of PD cases with coenzyme Q10 deficiency was also significantly greater in cases than in controls (32–36% versus 8-9%; *P* = 0.0012–0.006) [37]. Coenzyme Q10 has been very successful in preclinical models and early human studies [38], although failed in a large multicenter, double-blind, placebo-controlled, randomized clinical trial [39]. A recent meta-analysis of randomized controlled trials failed to demonstrate any improvement in PD motor symptoms following coenzyme Q10 supplementation [40]. The improvement in PD progression in this pragmatic survey that lost statistical significance when income was considered suggests that either individual in higher-income brackets is wasting their money on the ineffective coenzyme Q10 supplement or that higher income provides access to more neuroprotective nutrients and therapies. If the latter is true, the study design of controlled trials should be re-evaluated, as the artificial environment of controlled trials, for example, dopamine restriction, or the outcome measure, for example, UPDRS, may not be well suited to evaluating the effectiveness of coenzyme Q10 in a real-life setting.

Fish oil is a rich source of the omega-3 fatty acids eicosapentaenoic acid (EPA) and docosahexaenoic acid (DHA). There has been a tremendous amount of research conducted on the role of EPA and DHA in neuronal health. The neuroprotective effects of DHA, in particular, have been attributed to multiple mechanisms. In addition to acting as an antioxidant, DHA reduces inflammation by reducing arachidonic acid and its metabolites. As a precursor to neuroprotectin D1, it exerts antiapoptotic activity, enhances the synthesis of the neurotrophic factor, brain-derived neurotrophic factor (BDNF), and promotes neurogenesis via enhanced synaptogenesis and neurite outgrowth [41–43]. Only one randomized clinical trial has been conducted in which fish oil supplements were given to individuals with PD thus far, specifically targeting depression. In this pilot study, 31 individuals were randomized to fish oil capsules or mineral oil capsules for 12 weeks. At the end of the intervention period, those randomized to the fish oil capsules had statistically significant improvements in depression over the group randomized to mineral oil, as measured by the Montgomery-Asberg Rating Scale and Clinical Global Impression-Depression score, although this benefit was not apparent using the Beck Depression Inventory [44].

**Figure 2:**
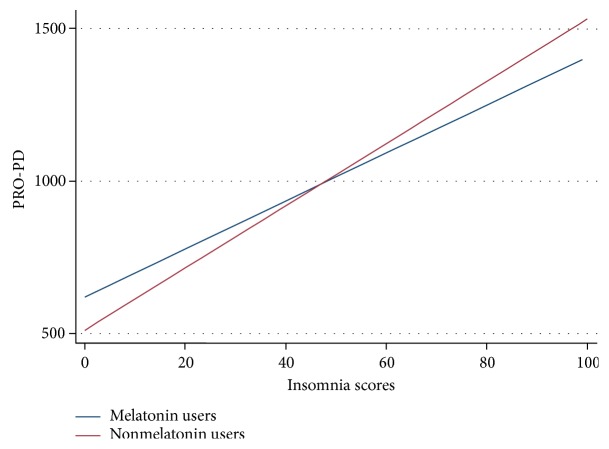
Correlation between insomnia and PD severity among individuals who do, and do not, report consistent use of melatonin over the previous six months.

There is conflicting evidence regarding the use of melatonin, a hormone produced by the pineal gland, in PD. Melatonin regulates the body's circadian rhythm: levels increase at night in response to the absence of light. The presence of this hormone induces the neurophysiological changes that occur during the brain restoration taking place during sleep. Sleep disorders are common in PD [45], and a substantial body of literature exists related to the neuroprotective role of melatonin, as well as to its putative role in treating PD motor and nonmotor impairments, including insomnia, anxiety, depression, and cognitive impairment [46]. A recent meta-analysis of randomized controlled clinical trials of exogenous melatonin for sleep disorders in neurodegenerative diseases found that melatonin improved rapid eye movement (REM) sleep behavior disorder (RBD) and Pittsburgh Sleep Quality Index (PSQI), although there was no evidence of improvement in objective sleep outcomes [47]. Thus, these data suggesting that melatonin use was associated with PD progression were at odds with the hypothesis that melatonin may stave off PD progression (Table 3). Because insomnia is also associated with PD progression and individuals with impaired sleep are more likely to take melatonin than those without sleep disorders, we observed whether the association remained after adjusting for impaired sleep (Figure 2). Insomnia and PRO-PD scores were correlated, and after adjusting for self-reported insomnia, the association between melatonin and PD progression was no longer significant (*P* = 0.001 and *P* = 0.406, resp.). These data suggest that insomnia, not the use of melatonin, is associated with PD progression.

Iron, prone to oxidation, has long been implicated in PD, and these results suggest that iron supplementation is associated with PD progression (Table 3). It is thought that the high iron content of the substantia nigra, required for dopamine synthesis, contributes to the selective vulnerability of the region. As already discussed, red meat consumption is associated with PD progression, while other high-fat meats, for example, pork, were not; it is possible that the high iron content of red meat may explain this correlation.

### 3.3. Nutritional Behaviors

After adjusting for age, gender, years since diagnosis, and income, individuals who prepare their own meals, and meals for others, were afforded protection against PD progression (Table 4). Individuals who report purchasing food from a local farmer's markets and going out of their way to eat organically grown food were also more likely to have lower PRO-PD scores (Table 4). This line of questions was designed to be a surrogate for mindfulness and attention to ingredients.

Individuals who find it difficult to afford food, especially healthy food, were associated with a faster rate of PD progression. While type 2 diabetes increases risk of PD [48], the association between the body weight index and risk of PD has been less clear [49].

## 4. Conclusions

This pragmatic, natural history study offers the first evidence-base for prescribing lifestyle modification (beyond exercise) to patients with PD. The foods shown here to be associated with slower PD progression are common to the Mediterranean diet and support an existing body of literature. Whether iron, beef, dairy, fried foods, diet soda, or canned goods provide environmental insults that accelerate disease progression warrants immediate attention; until further research is conducted, minimizing exposure to these foods is justified.

Because weight loss commonly occurs as the disease progresses, any suggestion that patients avoid foods increases the risk of restricting calories and contributing to malnutrition. Patients should be counseled on alternative sources of protein (e.g., beans, nuts, and seeds) and calcium (e.g., almonds, green leafy vegetables, and tofu).

Fish oil supplementation is warranted in individuals with a diagnosis of PD and justified based on biological plausibility and the clinical epidemiological data. As fish oil supplements are sold over the counter, there is a tremendous amount of diversity in both content of EPA and DHA, as well as in quality (e.g., presence of contaminants), whereas providers should familiarize themselves with available products and recommend buying from companies that perform analysis on stability, purity, and potency. Consumption of nonfried oily fish, such as herring, sardines, mackerel, and salmon should be encouraged. Because the association between coenzyme Q10 and PD progression was no longer significant after adjusting for income, more research needs to be done before recommending patients to start supplementing coenzyme Q10.

Health care providers should routinely review patients' supplement lists and ensure that the only patients taking iron are those with iron-deficiency anemia being treated under medical supervision. Because iron is commonly added to multivitamins, men and nonmenstruating women should not take a multivitamin containing iron, unless recommended by their physician.

The risk of bias should be considered in interpreting these data. Dietary intake is difficult to estimate (e.g., ingredients in casserole) and susceptible to recall bias (e.g., tasting food while cooking, food samples at the store, and other snacking are often under-reported). This is offset in this study because the questionnaire only asked about recent intake, the prior six months, and that all subjects in the study are affected equally by this limitation in recall. There is likely to be selection bias in this sample, as individuals using integrative medicine are expected to be more likely to enroll in a study called “Complementary and Alternative Medicine (CAM) in PD”. In an attempt to minimize this bias, the study home page explains that all individuals are invited to participate, regardless of disease duration, severity, or CAM use. Still, the degree to which these data are generalizable to the larger PD population has yet to be determined.

These data would be substantially improved with associated biomarkers of nutrient intake and physical examination of participants, both to screen for evidence of nutritional deficiencies and to confirm the diagnosis. This study is also limited by the homogeneity of the population, which was largely Caucasian and from the United States. Despite these limitations, these data suggest that the survey study design and PRO-PD are useful tools for deriving information about food, nutrition, and PD progression. As this is a longitudinal study that is still enrolling participants, it is likely that the foods and supplements associated with PD progression will change over time, as more people enroll, with evaluation of the longitudinal data. Clinicians now have data on which to base their recommendation for healthy eating in PD, and patients are likely to be empowered to know that their day-to-day choices may influence progression.

## Figures and Tables

**Table 1:** Demographics of the study participants.

	*N* = 1053
Age, years (SD)	63.1 (9.2)
Years since diagnosis (SD)	5.2 (5.5)
*Gender* Male Female	463 (44%) 556 (53%)
*Ethnicity* Caucasian Black Hispanic Native American Asian/Pacific Islander Other	978 (92.9%) 7 (0.7%) 14 (1.3%) 2 (0.2%) 9 (0.9%) 12 (1.1%)
*Income* Less than $20,000 Between $20 and 40,000 Between $40 and 60,000 Between $60 and 80,000 Between $80 and 100,000 Between $100 and 150,000 More than $150,000	56 (5.3%) 148 (14.1%) 139 (13.2%) 145 (13.8%) 137 (13%) 193 (18.3%) 155 (14.7%)
*Hoehn & Yahr stage* (1) 1-sided symptoms, minimal disability (2) Both sides affected, balance is stable (3) Mild-to-moderate disability, balance affected (4) Severe disability, able to walk and stand without help (5) Confinement to bed or wheelchair unless aided Do not know	522 (49.6%) 171 (16.2%) 292 (27.7%) 34 (3.2%) 2 (0.2%) 9 (0.9%)

**Table 2:** Multiple linear regression model of dietary intake and PD progression. Predicted PD severity score, as measured by the PRO-PD, per unit increase in food intake frequency, intake measured on a 10-point scale: never, <1/month, 1/month, 2-3×/month, 1/week, 2–4×/week, 5-6×/week, 1/day, 2–4×/day, 5-6×/day. ^∗^Adjusted for years since diagnosis, age, and gender. ^∗∗^Adjusted for years since diagnosis, age, gender, and income.

Association between dietary practices and Parkinson's disease progression				
Food item (serving size)	Mean change in PRO-PD score (SE)^∗^	*P* value (95% CI)^∗^	Mean change in PRO-PD score (SE)^∗∗^	*P* value (95% CI)^∗∗^
Fresh vegetables (1/2 cup)	−53.2 (7.9)	<0.000 (−68.7 to −37.6)	−48.9 (8.3)	<0.000 (−64.7 to −33.1)
Fresh fruit (1/2 cup)	−44.1 (8.5)	<0.000 (−60.7 to −27.5)	−40.7 (8.6)	<0.000 (−57.5 to −23.9)
Nuts (1/4 cup or 2 tbsp spread)	−38.5 (7.5)	<0.000 (−53.2 to −23.7)	−33.2 (7.6)	<0.000 (−48.1 to −18.4)
Fish (4 oz)	−37.1 (8.9)	<0.000 (−54.6 to −19.5)	−29.5 (9.1)	0.001 (−47.3 to −11.6)
Olive oil (1 tsp)	−34.1 (6.8)	<0.000 (−47.4 to −20.8)	−31.4 (6.8)	<0.000 (−44.7 to −18.1)
Wine (6 oz)	−23.6 (5.3)	<0.000 (−34.1 to −13.1)	−14.6 (5.6)	0.009 (−25.5 to −3.7)
Turkey (4 oz)	−20.2 (18.7)	0.281 (−57.1 to 16.7)	−10.8 (19.2)	0.573 (−48.7 to 27)
Coconut oil (1 tsp)	−18.6 (5.5)	0.001 (−29.3 to −7.8)	−20.2 (5.5)	<0.000 (−31 to −9.4)
Fresh herbs (1 tsp)	−14.9 (6.4)	0.02 (−27.4 to −2.4)	−8.9 (6.5)	0.169 (−21.7 to 3.8)
Spices (1/4 tsp)	−14.2 (6.4)	0.027 (−26.7 to −1.6)	−13.4 (6.4)	0.037 (−26 to −0.8)
Eggs (1 egg)	−9.5 (8.2)	0.251 (−25.6 to 6.7)	−9.7 (8.3)	0.241 (−26 to 6.5)
Bread (1 slice)	−7.7 (6.8)	0.26 (−21.2 to 5.7)	−6.9 (6.9)	0.314 (−20.4 to 6.6)
Beans (1/2 cup)	−6.3 (8.6)	0.466 (−23.3 to 10.7)	−5.4 (8.8)	0.54 (−22.6 to 11.8)
Butter (1 tsp)	−4 (5.9)	0.494 (−15.6 to 7.5)	−3.8 (6)	0.522 (−15.5 to 7.9)
Oatmeal (1 cup)	−3.2 (6.5)	0.624 (−15.9 to 9.5)	−4.4 (6.6)	0.501 (−17.3 to 8.5)
Liquor (1 oz)	−2.8 (7.7)	0.717 (−17.8 to 12.3)	3.6 (7.7)	0.47 (−11.5 to 18.7)
Green tea (1 cup)	−2.3 (5.7)	0.68 (−13.5 to 8.8)	1.6 (5.7)	0.779 (−9.6 to 12.7)
Juice (8 oz)	−2.3 (5.8)	0.687 (−13.8 to 9.1)	−1.4 (5.9)	0.811 (−12.9 to 10.1)
Frozen fruit (1/2 cup)	−1.9 (6.1)	0.757 (−13.8 to 10)	−2.2 (6.1)	0.714 (−14.1 to 9.7)
Cream (1/4 cup)	−0.5 (7.4)	0.942 (−15.2 to 14.1)	−0.3 (7.4)	0.971 (−14.7 to 14.2)
Coffee (8 oz)	−0.1 (4.4)	0.983 (−8.8 to 8.6)	4.3 (4.5)	0.342 (−4.5 to 13.1)
Soy (3 oz)	0.4 (7.9)	0.962 (−15.2 to 16)	2.3 (8)	0.77 (−13.4 to 18.1)
Safflower oil (1 tsp)	0.7 (6.9)	0.922 (−12.8 to 14.2)	6.8 (6.9)	0.325 (−6.8 to 20.5)
Beer (12 oz)	1.1 (7.6)	0.88 (−13.7 to 16)	2 (7.5)	0.789 (−12.8 to 16.8)
Chicken (4 oz)	3.3 (9.7)	0.34 (−15.6 to 22.3)	13.4 (9.8)	0.171 (−5.8 to 32.5)
Milk (1 cup) (mammalian, for example, cow)	5.8 (4.8)	0.226 (−3.6 to 15.2)	5.1 (4.8)	0.291 (−4.4 to 14.5)
Pork (4 oz)	6.1 (8.6)	0.482 (−10.8 to 22.9)	7 (8.7)	0.42 (−10 to 24)
Black tea (1 cup)	8.6 (5.6)	0.121 (−2.3 to 19.5)	8.4 (5.6)	0.131 (−2.5 to 19.3)
Eat food from a can	9.6 (8.1)	0.234 (−6.2 to 25.4)	6.1 (8.1)	0.449 (−9.7 to 22)
Pasta (1 cup)	10.1 (9.3)	0.28 (−8.2 to 28.4)	9.2 (9.4)	0.326 (−9.2 to 27.6)
Frozen vegetables (1/2 cup)	11 (6.9)	0.11 (−2.5 to 24.4)	10.3 (6.9)	0.137 (−3.3 to 23.9)
Cheese (1 slice, 1/2 oz, 1 tbsp)	11.7 (6.9)	0.091 (−1.9 to 25.3)	15.5 (6.9)	0.026 (1.9 to 29.1)
Yogurt (3/4 cup)	13.5 (7.5)	0.073 (−1.3 to 28.3)	15.2 (7.6)	0.046 (0.2 to 30.1)
Ice cream (1/2 cup)	13.8 (7.4)	0.064 (−0.8 to 28.3)	18.3 (7.5)	0.015 (3.6 to 32.9)
Soda (12 oz)	15.4 (7.8)	0.049 (0.03 to 30.7)	15.2 (7.9)	0.054 (−0.3 to 30.6)
Beef (4 oz)	16.2 (8.3)	0.051 (−0.1 to 32.4)	21.8 (8.3)	0.009 (5.5 to 38.1)
Fried food (4 oz)	19.5 (8.8)	0.027 (2.2 to 36.8)	23 (8.9)	0.009 (5.6 to 40.4)
Canned vegetables (1/2 cup)	19.9 (7)	0.005 (6.1 to 33.6)	18.3 (7)	0.009 (4.5 to 32.1)
Diet soda (12 oz)	20.7 (6.1)	0.001 (8.7 to 32.8)	23.6 (6.1)	<0.000 (11.6 to 35.6)
Canned fruit (1/2 cup)	36.1 (7.9)	<0.000 (20.5 to 51.6)	32 (7.9)	<0.000 (16.5 to 47.6)

**Table 3:** Logistic regression model of nutritional supplements and PD progression. Predicted PD severity score, as measured by the PRO-PD, based on the positive report of consistently using of supplements over the previous 6 months. ^∗^Adjusted for years since diagnosis, age, and gender. ^∗∗^Adjusted for years since diagnosis, age, gender, and income.

Association between dietary supplements & risk of Parkinson's disease progression					
Nutritional supplement	*n*	Mean change in PRO-PD score (SE)^∗^	*P* value (95% CI)^∗^	Mean change in PRO-PD score (SE)^∗∗^	*P* value (95% CI)^∗∗^
Inosine	13	−181.1 (125.6)	0.15 (−427.5 to 65.3)	−107.1 (122.9)	0.384 (−348.4 to 134.2)
Glutathione, oral	43	−126.1 (69)	0.068 (−261.6 to 9.3)	−126.7 (70)	0.07 (−263.9 to 10.5)
DHEA	47	−87.6 (70.8)	0.216 (−226.6 to 51.4)	−72.2 (70.9)	0.309 (−211.3 to 67)
Lithium, low dose	21	−84.9 (100.2)	0.397 (−281.6 to 111.8)	−118.9 (100.4)	0.237 (−315.9 to 78.1)
Low-dose naltrexone	14	−76.1 (120.9)	0.529 (−313.4 to 161.2)	−87.8 (118)	0.457 (−319.3 to 143.8)
CoQ10	286	−70.4 (31.5)	0.026 (−132.2 to −8.6)	−46.6 (31.6)	0.141 (−108.7 to 15.4)
Fish oil	376	−69.5 (29.5)	0.019 (−127.4 to −11.6)	−57.7 (29.6)	0.052 (−115.7 to 0.4)
Quercetin	21	−50.7 (105.9)	0.632 (−258.5 to 157.1)	−60.5 (106.4)	0.569 (−269.3 to 148.2)
Turmeric/curcumin	197	−47.3 (35.6)	0.186 (−117.3 to 22.8)	−49.5 (35.9)	0.168 (−120 to 20.9)
*Gingko biloba*	30	−47.2 (83.2)	0.57 (−210.5 to 116)	−61.1 (81.2)	0452 (−220.5 to 98.2)
Coconut oil	190	−35.8 (36.4)	0.324 (−107.2 to 35.5)	−52.7 (36.4)	0.147 (−124.1 to 18.6)
Resveratrol	43	−28.5 (70.7)	0.687 (−167.3 to 110.3)	−18.7 (72.7)	0.797 (−161.4 to 124)
Vitamin D	623	−26.1 (29)	0.368 (−83 to 30.8)	−3.6 (29.2)	0.902 (−60.9 to 53.7)
Alpha-lipoic acid	79	−19.1 (53.4)	0.72 (−123.9 to 85.7)	0.05 (54.4)	0.999 (−106.7 to 106.7)
5-Methyltetrahydrofolate (5-MTHF)	27	−17.1 (91.4)	0.852 (−196.4 to 162.2)	−25.1 (95.6)	0.793 (−212.7 to 162.5)
Probiotics	249	−12.3 (32.7)	0.708 (−76.5 to 52)	−12.4 (32.9)	0.706 (−77 to 52)
NADH	14	−9.7 (120.8)	0.936 (−246.7 to 227.3)	−25.2 (122.6)	0.837 (−265.7 to 215.4)
Multivitamin/mineral	342	−7.8 (30.2)	0.795 (−67.1 to 51.4)	9.9 (30.3)	0.744 (−49.6 to 69.5)
Calcium	324	−6.2 (32.2)	0.847 (−69.4 to 57)	12.5 (32.6)	0.701 (−51.4 to 76.4)
B6, B12, folic acid, betaine combination	88	3.4 (49.7)	0.946 (−94.2 to 101)	11.1 (48.9)	0.82 (−84.9 to 107.1)
Vitamin C	327	4.2 (30.6)	0.891 (−55.9 to 64.3)	−3.8 (31)	0.902 (−64.6 to 56.9)
N-Acetyl cysteine (NAC)	59	12.8 (60.1)	0.831 (−105 to 130.7)	26.9 (60.8)	0.658 (−92.4 to 146.1)
Vitamin B12 (methyl-B12/cyano-B12)	353	26.7 (29.8)	0.37 (−31.8 to 85.3)	43 (29.8)	0.15 (−15.6 to 101.6)
Rubidium	2	34.2 (306)	0.911 (−566.4 to 634.7)	93.1 (298.5)	0.755 (−492.7 to 678.8)
Estrogen	51	40 (67.4)	0.553 (−92.2 to 172.3)	15.2 (69.6)	0.827 (−121.4 to 151.8)
Glutathione, intranasal	24	62.9 (95.5)	0.51 (−124.5 to 250.4)	55.6 (93.2)	0.551 (−127.3 to 238.5)
*Mucuna*	33	67 (81.7)	0.412 (−93.3 to 227.2)	21.8 (80)	0.785 (−135.1 to 178.6)
Fava beans	17	122 (109)	0.263 (−92 to 336)	87.8 (110.1)	0.425 (−128.2 to 303.8)
Melatonin	148	139.3 (40.5)	0.001 (59.8 to 218.8)	134.8 (40.2)	0.001 (56 to 213.6)
Iron (Fe)	57	146.4 (63.9)	0.022 (21 to 271.9)	179.7 (64.3)	0.005 (53.6 to 305.9)

**Table 4:** Logistic regression model of dietary behaviors and PD progression. Predicted PD severity score, as measured by the PRO-PD. Participants were asked to select all of the statements that were true over the prior 6 months; plastic bottle utilization was evaluated on a 10-point scale, also over the prior 6 months. ^∗^Adjusted for years since diagnosis, age, and gender. ^∗∗^Adjusted for years since diagnosis, age, gender, and income.

Dietary behaviors associated with Parkinson's disease progression				
Dietary behaviors	Mean change in PRO-PD score (SE)^∗^	*P* value (95% CI)^∗^	Mean change in PRO-PD score (SE)^∗∗^	*P* value (95% CI)^∗∗^
I routinely prepare meals for others.	−141.1 (29.3)	<0.000 (−198.7 to −83.6)	−112.8 (29.7)	<0.000 (−171.2 to −4.4)
I cook most of my meals.	−115.1 (30.2)	<0.000 (−174.4 to −55.7)	−135.4 (30.3)	<0.000 (−194.9 to −5.8)
I buy food from a local farmers (co-op, farmer's markets)	−98 (28.3)	0.001 (−153.5 to −42.5)	−97.2 (28.4)	0.001 (−153 to −41.5)
I try to eat organically grown foods when possible.	−61.3 (28.1)	0.029 (−116.5 to −6.2)	−74.9 (28.1)	0.008 (−130 to −19.8)
I drink from a plastic bottle.	11.2 (5.4)	0.039 (0.6 to 21.8)	11.9 (5.4)	0.029 (1.2 to 22.6)
I am overweight.	169.4 (28.5)	<0.000 (113.5 to 225.3)	165.8 (28.5)	<0.000 (109.8 to 221.8)
It is difficult to afford groceries.	443.5 (51.2)	<0.000 (343.1 to 543.9)	348.7 (55.4)	<0.000 (240 to 457.3)
I find it difficult to afford healthy food.	473.6 (46.1)	<0.000 (383 to 564.1)	389.3 (49.5)	<0.000 (292.2 to 486.5)
